# Feasibility and long-term results of focused radioguided parathyroidectomy using a "low" 37 MBq (1 mCi) ^99m^Tc-sestamibi protocol

**DOI:** 10.1186/1477-7800-3-30

**Published:** 2006-09-15

**Authors:** Domenico Rubello, Adil Al-Nahhas, Giuliano Mariani, Milton D Gross, Lucia Rampin, Maria Rosa Pelizzo

**Affiliations:** 1 Nuclear Medicine Service, 'S. Maria della Misericordia'Hospital, Istituto Oncologico Veneto (IOV), Rovigo, Italy; 2 Nuclear Medicine Department, Hammersmith Hospital, London, UK; 3 Regional Centre of Nuclear Medicine, University of Pisa Medical School, Pisa, Italy; 4 Department of Radiology, Division of Nuclear Italy Medicine, University of Michigan and Department of Veterans Affairs Health System, Ann Arbor, Michigan, USA; 5 Department of Surgical Science, University of Padova Medical School, Padova, Italy

## Abstract

Aim of the present study was to investigate the feasibility and long-term results of focused radioguided parathyroidectomy using a "low" 37 MBq (1 mCi) ^99m^Tc-sestamibi dose protocol compared to conventional "high 740 MBq (20 mCi) ^99m^Tc-sestamibi dose protocol" in patients with primary hyperparathyroidism (PHPT). The data of focused radioguided surgery obtained in a group of 320 consecutive PHPT patients with high probability of the presence of a solitary parathyroid adenoma (PA) were studied. All patients underwent preoperative imaging work-up of double-tracer ^99m^Tc-pertechnetate/^99m^Tc-sestamibi subtraction parathyroid scintigraphy (Sestamibi scintigraphy) and high resolution neck ultrasound (US). In 301/320 patients (96.6%) focused minimally invasive radioguided surgery was successfully performed by administering a "low" 37 MBq (1 mCi) ^99m^Tc-sestamibi dose in the operating room 10 minutes before operation. No major intraoperative complications were recorded. Focused radioguided surgery required a mean time of 32 min and a mean hospital stay of 1.2 days. Local anesthesia was applied in 75 patients, 66 of whom (88%) were patients older than 65 years with comorbidities contraindicating general anesthesia. No case of persistent or recurrent PHPT was observed during post-surgical follow-up (range = 18–70 months; mean +/- SD = 15.3 +/- 9.1 months). Radiation exposure dose to the operating surgeon was 1.2 μSi/hour with the "low 37 MBq (1 mCi) ^99m^Tc-sestamibi dose", and less than 1.0 μSi/hour for the other operating-room personnel. Focused low dose radioguided parathyroidectomy is a safe and effective means to localize parathyroid adenomas in patients affected by solitary PA thus reducing by 20 fold the radiation exposure dose to the patients and operating room personnel.

## Background

The surgical approach to patients with primary hyperparathyroid (PHPT) has moved from bilateral neck exploration (BNE) to minimally invasive operative alternatives of endoscopic or focused radioguided surgery.

The popularity of the focused surgical approach has occurred as a result of technical improvements in surgical practice by the introduction of the endoscope, the intra-operative gamma probe, and the availability of rapid, intraoperative measurements of "quick" parathyroid hormone (QPTH). In a worldwide survey published in 2002, more than half of endocrine surgeons favored a minimally invasive approach to PHPT patients with a high likelihood of a solitary parathyroid adenoma (PA) as the etiology of their disease [1].

In contrast to the more traditional approach of BNE, the minimally invasive focused approach requires rapid and accurate preoperative localization:

• to establish the etiology of PHPT;

• to precisely localize PA (orthotopic or ectopic position, deepness, etc);

• to distinguish the presence of 99mTc-sestamibi-avid thyroid nodules mimicking PA and which may result in false positive intraoperative probe localization [3,4].

The present study details our experience with focused minimally invasive parathyroidectomy in a group of 300 consecutive patients using the "low 37 MBq (1 mCi) sestamibi protocol" and the intraoperative gamma probe.

## Materials and methods

Three-hundred and twenty consecutive patients with clinically and biochemically proven PHPT entered the study. There were 207 females and women and 113 men, mean age 55.3 years (range 16–83 years). Fifty-five, (55) of these patients had undergone in other centers previous thyroid or unsuccessful parathyroid surgery. The following inclusion criteria were used for focused radioguided, minimally invasive, parathyroidectomy: (a) the evidence at double-tracer ^99m^Tc-pertecnetate/^99m^Tc-sestamibi subtraction (^99m^Tc-sestamibi) scintigraphy of a solitary PA; (b) unambiguous ^99m^Tc-sestamibi uptake in the suspected PA; (c) absence of other ^99m^Tc-sestamibi-avid nodules; (d) absence of history of familial hyperparathyroidism or multiple endocrine neoplasia (MEN); or (e) no prior neck irradiation.

One hundred and seven patients were excluded from focused minimally invasive parathyroidectomy for not meeting these inclusion criteria and were offered traditional BNE.

Preoperative imaging consisted of a single-session ^99m^Tc-sestamibi scan and high resolution neck ultrasound (US) as previously described [3,5-8]. In patients with concordant ^99m^Tc-sestamibi/US findings (both positive or negative) no further imaging was performed, while in discrepant cases (^99m^Tc-sestamibi positive but US negative) a ^99m^Tc-sestamibi tomographic (SPECT) examination (n. = 111 patients) was also obtained to evaluate possible ectopic or deep-seated PA located in the para-retroesophageal/para-retrotracheal space. SPECT was obtained immediately after the completion of planar ^99m^Tc-sestamibi imaging and as a result, ^99m^Tc-sestamibi re-injection was not necessary, avoiding additional radiation exposure. The scintigraphic studies required approximately 1 hour (40 minutes for the planar and 20 minutes for SPECT imaging) to complete).

The scintigraphic images were interpreted by two nuclear medicine physicians; in cases of disagreement the final impression was reached by consensus. In patients with a normal thyroid gland confirmed both at ^99m^Tc-pertechnetate scintigraphy and neck US, a single focus of ^99m^Tc-sestamibi uptake was considered to be consistent with a solitary PA while in patients in whom at least two foci of ^99m^Tc-sestamibi uptake were demonstrated multiple adenomas were reported. In patients with concomitant ^99m^Tc-sestamibi positive thyroid nodules, neck US, ^99m^Tc-pertecnetate thyroid scintigraphy was used to distinguish PA from the sestamibi-avid thyroid nodule(s).

Planar sestamibi scintigraphy was acquired by a large-field-of-view (LFOV) gamma camera (Orbiter, 7500, Siemens, Hoffman Estates, IL or E-CAM, Siemens, Hoffman Estates, IL) equipped with a parallel hole, low-energy, high resolution collimator. Images were stored in a 128 × 128 matrix and processed using a dedicated computer.

Tomographic sestamibi SPECT acquisition was performed by a dual-head gamma camera (Axis, Picker International, Cleveland, Ohio or E-CAM, Siemens, Hoffman Estates, IL) equipped with a couple of parallel-hole low-energy ultra-high-resolution collimators. The following parameters were adopted: elliptical orbit, 120 (64 × 2) steps, 30 sec per step, 64 × 64 matrix. Images were reconstructed using a Butterworth filter, cut-off 0.45–0.65, order 5–8, and processed using a dedicated computer: three-dimensional (3D) analysis and £D rendering imaging was also obtained.

Neck ultrasound was performed using high resolution, 7.5–12.5 MHz, transducer (Technos, Esaote, Italy). Longitudinal and axial neck scans were obtained from the angle of the mandible to the sternal notch. The PA was identified on gray-scale imaging as a hypo-echoic nodule well distinct from the thyroid gland.

After operation, the surgeon was asked to judge the utility of the gamma probe during operation using a 4-point scale of "not valuable", "slightly valuable", "definitely valuable", and "very valuable".

The intraoperative technique used in our center for minimally invasive radioguided surgery is outlined in Table 1.

**Table 1:** Characteristics of our patients population affected by primary hyperparathyroidism

	**Patients in whom focused miminally invasive parathyroidectomy was planned**	**Patients in whom bilateral neck exploration was planned**
Total patients number	320	107
Solitary PA and normal thyroid gland*	320	0
Solitary PA and nodular goitre*	0	51
Parathyroid multigland disease	0	27
Negative sestamibi scan	0	21
History of familial hyperparathyroidism	0	3
History of multiple endocrine neoplasia (MEN)	0	4
History of neck irradiation	0	1
Previous thyroid/parathyroid surgery	75	18
Mean (and range) levels of preoperative serum calcium (mg/dl)	12.1 (10.6 – 13.8)	12.2 (10.6 – 13.7)
Mean (and range) levels of preoperative serum PTH (pg/ml)	196 (84 – 351)	221 (91 – 336)
Mean (and range) operating time, minutes	32 (15 – 58)	78 (52 – 107)
Mean (and range) hospital stay, days	1.2 (1 – 2)	2.5 (2 – 5)
Mean (and range) post-surgical follow-up, months	15.3 (18 – 70)	15.9 (18 – 83
Recurrent hyperparathyroidism	0 cases	4 cases; 3.7% (all multigland disease at first diagnosis)

PA = parathyroid adenoma

*Thyroid gland evaluated by thyroid scan and ultrasound

A hand-held commercially available collimated gamma probe (Scintiprobe MR 100, Pol.hi.tech., Italy) was used. Intraoperative QPTH was measured by immunochemoluminescent assay (Liason, Byk Gulden, Italy). A fall of 50% or more in PTH levels 10 minutes after PA removal in comparison with baseline pre-excision value was considered indicative of a successful parathyroidectomy. Additional blood samples for QPTH measurement were obtained in patients with multigland disease after removal of any hyperfunctioning parathyroid gland.

All operations were performed by the same surgeon (M.R.P.). Post-surgical follow-up ranged 18–70 months, mean +/- SD = 15.3 +/- 9.1 months.

All patients received clinical and laboratory surveys 1 month after surgery and subsequently every 2–3 months, thereafter.

Data are expressed as mean +/- 1 standard deviation (SD). Mean values were compared using Student's *t *test. *P *values lower than 0.05 were considered significant.

## Results

Charactheristics of our patient population are resumed in Table 2.

**Table 2:** Focused radioguided surgery using the 'low sestamibi dose' procedure.

*(a) *QPTH levels measured just before intervention and 10 min after PA removal
*(b) *37 MBq (1 mCi) of ^99m^Tc-sestamibi injected in the operating room 10 min before intervention
*(c) *prior to surgery, patient's neck scanned with the probe to individualize the PA
*(d) *a transverse midline neck 1.5 – 2 cm access, 1 cm above the sternal notch, is performed
*(e) *probe is repeatedly inserted through the wond guiding the surgeon to the PA.
*(f) *radioactivity is measured on the PA, thyroid gland and background
*(g) *radioactivity is measured on the *ex vivo *PA
*(h) *radioactivity is checked on the empty operating basin
*(i) *tissue ratios are calculated (P/B, P/T)

QPTH = quick parathyroid hormone

BNE = bilateral neck exploration

PA = parathyroid adenoma

P/B = parathyroid to background ratio

P/T = parathyroid to thyroid ratio

**Figure 1:**
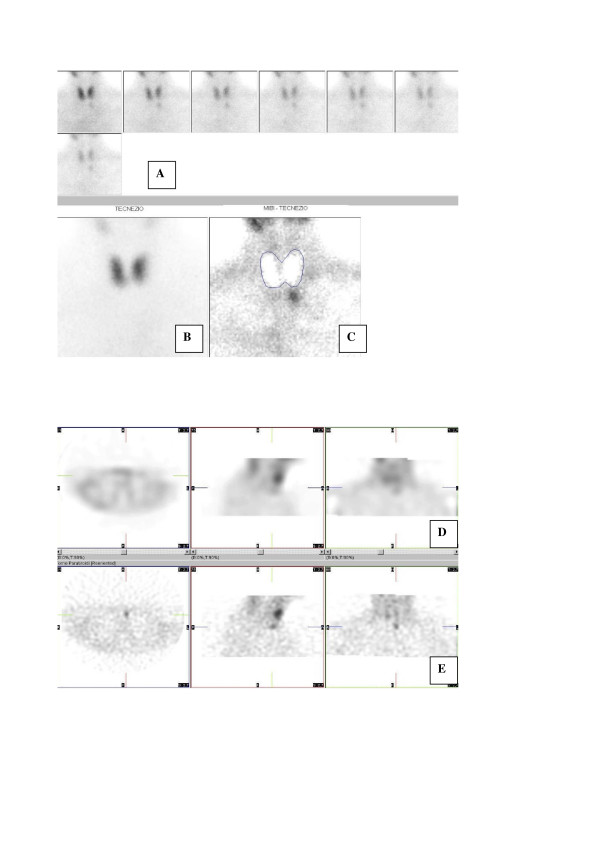
Scintigraphy showing a left inferior parathyroid adenoma located just beyond the thyroid left lobe shape, and in anterior planes at SPECT examination. A = sequential acquisition of a set of ^99m^Tc-sestamibi images, lasting 5 minutes each. B = ^99m^Tc-pertechenetate thyroid image. C = subtraction image showing a left inferior solitary parathyroid adenoma. D = Sestamibi SPECT images (left = axial; middle = sagittal; right = coronal) reconstructed by Butterworth filter with sub-optimal parameters (cut-off = 0.40, order = 7); E = Sestamibi SPECT images (left = axial; middle = sagittal; right = coronal) reconstructed by Butterworth filter with optimal parameters (cut-off = 0.60, order = 7) depicting a left inferior parathyroid adenoma located in anterior neck planes.

On the basis of preoperative ^99m^Tc-sestamibi scintigraphy and high resolution neck US results, focused minimally invasive parathyroidectomy was offered to 320 patients, and was successfully performed in 309 (96.6%). Figure 1 shows an example of double-tracer planar and SPECT sestamibi scintigraphy.

In 11 patients BNE was performed: in 2 patients because of suspected parathyroid carcinoma, in 4 patients because of a persistently elevated QPTH levels after removal of the preoperatively visualized PA (in all these 4 cases a second PA was found during subsequent BNE), and in 4 because of a technical difficulties due to either unusual position and/or large size precluded minimally invasive parathyroidectomy.

Considering the group of 309 patients in whom focused minimally invasive parathyroidectomy was successfully performed, the surgeon judged the gamma probe as "slightly valuable" in 29 cases (9.4%), "definitely valuable" in 203 cases (65.7%) and "very valuable" in 77 cases (24.9%). The probe was critical in identifying 21 ectopic PAs located in the mediastinum, 2 ectopic PAs located at the carotid bifurcation, and 24 PAs located deep in the neck in the para-retroesophageal/para-retrotracheal space. Mean operating time was 32 minutes (range, 15–64) and the mean hospital stay of 1.2 days (range, 1–2 days). Local anesthesia was successfully performed in 75 patients, the majority of whom (n. = 66; 88%) were older than 65 years with comorbidities contraindicating general anesthesia. No major surgical complication (laryngeal nerve palsy, permanent hypoparathyroidism) were noted. Transient hypocalcemia was observed in 8.6% of cases. A small 1.5 to 2 cm skin incision was sufficient to perform focused minimally invasive radioguided parathyroidectomy. It is worth noting that focused parathyroidectomy was successfully performed in 42/55 patients (76.4% of cases) with recurrent hyperpararthyroidism, prior thyroid surgery or previously unsuccessful parathyroid surgery.

The mean PA to background (P/B) uptake ratio was high (2.6); and the mean PA to thyroid (P/T) ratio was relatively elevated (1.5). Of note, normal parathyroid glands intraoperatively identified by the probe did not demonstrate 99m Tc-sestamibi accumulation over background.

In the group of patients in whom SPECT was performed, it is worth noting that in 39 cases SPECT analysis correctly suggested a deep location of PA which was confirmed at operation.

The mean weight of the excised solitary PA by MIRS was 990 +/- 310 mg. The parathyroid glands removed in patients with multiple gland disease were significantly smaller (mean weight = 530 +/- 420 mg; p < 0.05).

Mean radiation exposure in the operating room after the injection of a low 37 MBq (1 mCi) of ^99m^Tc-sestamibi dose, was 1.2 μSi/hour for the surgeon and less than 1.0 μSi/hour for other operating suite personnel. Present European Atomic Energy Community (EURATOM) recommends a radiation dose to the general public of < 1000 μSi/year, using this regulatory threshold, an endocrine surgeon and support operating room staff could perform approximately 1000 – 1500 focused parathyroidectomies per year with the "low 37 MBq (1 mCi) of ^99m^Tc-sestamibi dose" protocol before reaching the EURATOM recommended dose for the general population.

## Discussion

Tibblin et al. made the first attempt to perform a limited neck exploration in PHPT patients in the early '80s [9]. They performed a unilateral neck exploration removing the PA with biopsy of the ipsilateral parathyroid gland in an attempt to identify the presence of glandular hyperplasia. More recently minimally invasive endoscopic [10,11] and gamma probe-guided [3-8,12-14] approaches were developed for focused minimally invasive parathyroidectomy.

The development and popularity of focused minimally invasive, parathyroidectomy, has occurred because of *(i) *the improvement in preoperative localization imaging mainly related to ^99m^Tc-Sestamibi scintigraphy [15-20] and *(ii) *the introduction in clinical practice of intraoperative QPTH measurements. Successful focused minimally invasive parathyroidectomy requires strict attention to patient selection criteria that include:

(a) high likelihood of a solitary PA demonstrated at preoperative ^99m^Tc-sestamibi/US imaging;

(b) unambiguous ^99m^Tc-sestamibi uptake in the PA;

(c) absence of other ^99m^Tc-sestamibi avid thyroid nodules.

Using these selection criteria about two thirds of all PHPT patients can be offered focused minimally invasive parathroidectomy as an alternative to BNE [3-8,12-14].

Thus, the success of focused parathyroidectomy is dependent upon precise preoperative localization of a solitary PA. In this regard, there is evidence that the combination of ^99m^Tc-sestamibi scintigraphy and high resolution neck US provides several diagnostic localization advantages:

(a) more accurate information about PA localization (orthotopic or ectopic site, deepness, etc)

(b) differentiation of solitary PA from multiple gland disease;

(c) evaluation of presence of co-existing nodular goiter [3-8,14].

(d) furthermore, the use of SPECT improves sensitivity and accuracy of the imaging procedure [22,23].

In our protocol, neck US was systematically combined with ^99m^Tc-sestamibi scintigraphy while SPECT was reserved for patients with ectopic parathyroid glands or incongruent ^99m^Tc-sestamibi (positive)/US (negative) results.

The first focused minimally invasive parathyroidectomy protocol was developed by Norman in USA in 1997 [12]: it consisted of a single-day, imaging and surgery approach, with the patient injected with a 740–925 MBq (20–25 mCi) of sestamibi and imaging obtained using a dual-phase technique with focused parathyroidectomy via a lateral neck access performed within 2–3 hours after radiotracer administration. Norman's protocol is attractive from a cost-analysis perspective because ^99m^Tc-sestamibi scintigraphy and focused surgery are performed on the same day using a single dose of ^99m^Tc-sestamibi that is sufficient for both planar scintigraphy and "focused' radioguided surgery. However, Norman's protocol presents practical disadvantages given the uncertainty of the scintigraphic results and differences between the minimally invasive parathyroidectomy and BNE with respect to the need for operating theatre time and efficient patient scheduling. Indeed, this problem would be expected to be even greater in geographic areas with a high prevalence of nodular goiter so that a multiple-day imaging protocol would be preferable [14]. The approach we use is a modified, multiple-day protocol: the first day imaging is obtained using dual-tracer ^99m^Tc-pertecnetate/^99m^Tc-sestamibi subtraction scintigraphy combined with high resolution neck US. The day of focused radioguided surgery is usually performed within 1 week of imaging, using a low 37 MBq (1 mCi) ^99m^Tc-sestamibi dose administered in the operating room 10 minutes before operation. The 'low sestamibi dose protocol' we use provides the two main advantages of (a) less radiation exposure to the patient and operating suite personnel (approximately 20-fold lower than the "high sestamibi dose protocol" and (b) fewer false negative results in PA exhibiting rapid ^99m^Tc-sestamibi wash-out [24].

Favorable results have been reported with both with Norman's 'high sestamibi dose' protocol and our 'low sestamibi dose' protocol with a success of intraoperative detection of PA of 95% or more, without major intraoperative surgical complications. It is reasonable to assume that Norman's single-day protocol would be preferable in patients with a low-likelihood of nodular goiter while the multiple-day protocol would be preferable in areas with a high prevalence of nodular goiter.

Another advantage of focused radioguided surgery is the ability to perform minimally invasive neck exploration under local anesthesia with early (also same-day) hospital discharge [14]. Although attractive we prefer to offer this approach to elderly and medically complex patients in whom general anesthesia would contraindicated with planned hospital on the 2^nd ^postoperative day [25,26].

In conclusion, focused minimally invasive surgery is a safe and effective approach for PHPT patients with a high probability of a solitary PA. Preoperative accurate imaging, based on ^99m^Tc-sestamibi scintigraphy, is necessary. Our data confirm that a low 37 MBq (1 mCi) ^99m^Tc-sestamibi dose administered in the operating room 10 minutes before surgery is sufficient to successfully perform a focused parathyroidectomy.
